# F168. PSYCHOTIC EXPERIENCE AND ADOLESCENT BRAIN TRAJECTORY: EVIDENCE FOR STRUCTURAL ALTERATIONS IN DOPAMINERGIC REGIONS

**DOI:** 10.1093/schbul/sby017.699

**Published:** 2018-04-01

**Authors:** Andre Zugman, Ary Gadelha, Felipe Argolo, Pedro Pan, Luis Augusto Rohde, Euripedes C Miguel, Rodrigo Bressan, Andrea Parolin Jackowski

**Affiliations:** Federal University of Sao Paulo

## Abstract

**Background:**

Psychotic Experiences (PE) are often reported by children and adolescents and have a bidirectional association with mental disorders, both increasing subsequent risk for mental disorders and being more frequently reported among subjects with a current psychiatric diagnosis. The brain developmental trajectories associated with PE in children and adolescents and how PE may evolve to a full blown mental disorder remain largely unknown. We assessed PE effect on subcortical and cortical measures over a 3-year follow-up in a community cohort of children and adolescents.

**Methods:**

This study is part of the Brazilian High-risk Cohort, a multi-site longitudinal study. A total of 2,512 youths (6–12 years old, mean age at baseline 9.7 years, SD = 1.92; 53,1% male) completed the baseline assessment and 2,012, the 3-year follow-up (T1). PE were assessed at the two time-points with The Community Assessment of Psychic Experience (CAPE). A confirmatory factor analysis (CFA) was used to generate a latent score for PE for baseline and follow-up, yielding good model fits.

A subset of the sample (n=809) was scanned in a 1.5T scanner on either baseline or follow-up resulting in 1183 MRI scans. Structural images were processed using Freesurfer. Subcortical volumes for the amygdala, hippocampus, caudate, putamen and pallidum were entered as a dependent variable in a linear mixed effects model (lme) with age, sex, CAPE and CAPE by age interaction as fixed effects and site and subject as random effects. The same model was applied in a mass-univariate analysis for cortical thickness measure (CT). A smoothing kernel (FWHM = 10 mm) was applied to CT before statistical testing. False Discovery Rate was used to control for multiple comparisons in the mass-univariate analysis with a p<0.05 threshold.

**Results:**

CAPE was significantly related to the right putamen (Beta: -0.30 p:0.03), right caudate (Beta: -0.32 p: 0.03) and left caudate (Beta: -0.32 p:0.02). The age by CAPE interaction was significant for the three regions (right putamen Beta: 0.001 p:0.04, right caudate Beta:0.002 p: 0.03 and left caudate Beta: 0.001 p:0.03).

CAPE and CAPE by age interaction terms showed no effect on cortical thickness after correction for multiple comparisons.

**Discussion:**

PE report was associated with lower subcortical volumes of the caudate and putamen, regions of the striatum. The striatum receives important dopaminergic neurotransmission and have been previously implicated in the pathogenesis of schizophrenia. In line with the hypothesis in schizophrenia, the PE experienced by children and adolescents may relate to a dopaminergic imbalance, possibly due to developmental structure alterations of the striatum and its connections. Interestingly, neither the hippocampus nor the amygdala were related to PE. Taken together with the lack of findings related to cortical thickness, the results presented here suggests a probable dopaminergic role on PE in young people with no current psychotic disorder.

